# APOBEC3DE Inhibits LINE-1 Retrotransposition by Interacting with ORF1p and Influencing LINE Reverse Transcriptase Activity

**DOI:** 10.1371/journal.pone.0157220

**Published:** 2016-07-18

**Authors:** Weizi Liang, Jiwei Xu, Wensu Yuan, Xuan Song, Jianyong Zhang, Wei Wei, Xiao-Fang Yu, Ying Yang

**Affiliations:** 1 School of Life Sciences, Tianjin University, Tianjin, China; 2 First Hospital of Jilin University, Institute of Virology and AIDS Research, Changchun, Jilin Province, China; 3 Department of Molecular Microbiology and Immunology, Johns Hopkins Bloomberg School of Public Health, 615 N. Wolfe Street, Baltimore, MD 21205, United States of America; Institute of Medicinal Biotechnology, Chinese Academy of Medical Sciences, CHINA

## Abstract

Human long interspersed elements 1 (LINE-1 or L1) is the only autonomous non-LTR retroelement in humans and has been associated with genome instability, inherited genetic diseases, and the development of cancer. Certain human APOBEC3 family proteins are known to have LINE-1 restriction activity. The mechanisms by which APOBEC3 affects LINE-1 retrotransposition are not all well characterized; here, we confirm that both A3B and A3DE have a strong ability to inhibit LINE-1 retrotransposition. A3DE interacts with LINE-1 ORF1p to target LINE-1 ribonucleoprotein particles in an RNA-dependent manner. Moreover, A3DE binds to LINE-1 RNA and ORF1 protein in cell culture system. Fluorescence microscopy demonstrated that A3DE co-localizes with ORF1p in cytoplasm. Furthermore, A3DE inhibits LINE-1 reverse transcriptase activity in LINE-1 ribonucleoprotein particles in a cytidine deaminase-independent manner. In contrast, A3B has less inhibitory effects on LINE-1 reverse transcriptase activity despite its strong inhibition of LINE-1 retrotransposition. This study demonstrates that different A3 proteins have been evolved to inhibit LINE-1 activity through distinct mechanisms.

## Introduction

The apolipoprotein B mRNA-editing catalytic polypeptide 3 (APOBEC3) proteins are cytidine deaminases related to AID (activation-induced cytidine deaminase) and APOBEC1 (apolipoprotein B mRNA editing enzyme, catalytic polypeptide1). The APOBEC3 (A3) family consists of seven family members (APOBEC3A, -B, -C, -DE, -F, -G, and–H) with diverse activities against a variety of retroviruses and endogenous retroelements. Certain A3 family members can potently suppress retrovirus replication by editing the viral genome during reverse transcription via cytidine deamination as well as other mechanisms [1–7]. In order to successfully replicate, HIV-1 encodes Vif to inactivate some of these A3 molecules. Vif hijacks the host E3 Cul5 ubiquitin ligase system to induce polyubiquitination and degradation of the A3 molecules [3, 7–14].

The underlying mechanism governing these diverse anti-viral activities of the A3 proteins is still not clear. The anti-viral effects of the most potent HIV-1 restriction factors, A3G, A3H, and A3F, require efficient packaging into the HIV-1 virions [5, 15–18]. In the absence of Vif, the incorporation of A3G and A3F into virions requires the RNA binding nucleocapsid (NC) domain of Gag and viral and/or cellular RNAs [15, 16, 18–20]. In newly infected target cells, virion-packaged A3 proteins induce cytidine deamination of viral cDNA and block the completion of full-length viral DNA during reverse transcription. A3 proteins can also inhibit the nuclear importation of HIV-1 and the integration of viral DNA.

In addition to retroviral inhibition, various A3 family members have been shown to inhibit the human non-LTR retrotransposon LINE-1 to varying degrees [21]. LINE-1 is the only active autonomous retroelement in humans and makes up at least 17% of the human genome [22–25]. LINE-1 can also mediate the retrotransposition of other non-autonomous retroelements, such as Alu and SVA, which account for more than 20% of the human genome [26, 27]. Ninety-seven human diseases are related to germ-line insertions of endogenous retrotransposons [24].

Human LINE-1s are transcribed by RNA polymerase II to mRNAs of about 6 kb that contain a 5’ untranslated region (UTR), two open reading frames (ORF1 and ORF2), and a 3’ UTR [28]. ORF1 encodes a RNA-binding protein (ORF1p), and ORF2 encodes a protein (ORF2p) with endonuclease and reverse transcriptase activities [29]. Both ORF1p and ORF2p are required for retrotransposition during target site-primed reverse transcription (TPRT). The LINE-1 replication cycle proceeds as follows: (i) LINE-1 DNA is transcribed into mRNA in the nucleus and transported to the cytoplasm, then translated into the ORF1 and ORF2 proteins; (ii) the LINE-1RNA assembles into ribonucleoprotein (RNP) particles with ORF1p and ORF2p; (iii) LINE-1 RNP is transported to the nucleus and integrated into the genome through TPRT [30].

Besides APOBEC3G, other well characterized anti-retrovirus factors such as MOV10, SAMHD1, ZAP have been identified to be able to inhibit LINE-1 retrotransposition through diverse mechanisms. MOV10, a helicase inhibits LINE-1 mobility through interacting with LINE-1 RNP and causing LINE-1 RNA degradation [31]. SAMHD1 can inhibit the L1 ORF2p RT activity and promote cellular stress granule assembly, while there is no detectable binding of SAMHD1 with LINE-1 RNA. [32]. ZAP can restrict LINE-1 mobility. It reduces the transcription of LINE-1 RNA and the expression of LINE-1 protein, and interacts with ORF1p in a RNA-dependent manner. [33]. These accumulated evidences suggested that host antiviral restriction factors using distinct mechanisms to antagonize exogenous retrovirus and endogenous retroelements [34]

Unlike A3-mediated HIV-1 restriction, the mechanism of A3-mediated LINE-1 inhibition has not been well characterized. A3A is a potent LINE-1 inhibitor, and its inhibitory function requires its deaminase domain and deaminase activity. A3A can induce cytidine deamination of LINE-1 DNA. On the other hand, several A3 proteins can mediate LINE-1 inhibition in the absence of a functional deaminase domain [35, 36]. It is generally believed that A3G has weak or no activity against LINE-1 retrotransposition, and the effect of A3F on LINE-1 retrotransposition is controversial [6, 36–39].

In the present study, we demonstrate that both human A3B and A3DE are potent inhibitors of LINE-1 retrotransposition but have different LINE-1 RNP-targeting activities. A3DE binding to LINE-1 ORF1p is RNA-dependent, implying an interaction between A3DE and LINE-1 ribonucleoprotein particles (RNPs). Furthermore, the amount of LINE-1 cDNA synthesized by LINE-1 RT is significantly reduced by A3DE but not by A3B. The restrictive activity of A3DE on LINE-1 is independent of its cytidine deaminase activity. Our study demonstrates that the human APOBEC3 restriction factors inhibit LINE-1 retrotransposition through diverse pathways.

## Materials and Methods

### Cells and Plasmids

HEK293T cells(293T/17 [HEK 293T/17] (ATCC® CRL11268™)) were cultured in DMEM medium with supplement of 10% FBS (GIBCO) and 1% Penicillin-Streptomycin stock solution (Invitrogen, 5,000unit Penicillin and 5,000 μg Streptomycin per milliliter). The expression vectors of HA/V5-tagged A3B and A3DE were constructed in our laboratory. HA-tagged A3DE-E80Q, A3DE-E264Q, A3DE- E80Q/E264Q and A3B-W228L/ D316N were generated by overlapping PCR and confirmed by sequencing. The human L1 plasmids 99 PUR L1RP EGFP (L1) and 99 PUR JM111 EGFP (JM111, an L1 construct containing two point mutations in ORF1, which cause complete abolishment of LINE-1 retrotransposition, was used as a negative control) were gifted from Professor Kazazian HH Jr and had been described previously [40]. The pcDNA3.1-EGFP plasmid was cloned in our laboratory.

### Cell Viability Assay

The empty vector, the A3B-HA, or the A3DE-HA expression vector was co-transfected with the pcDNA3.1 EGFP separately into HEK293T cells in 12-well plates. The cells were harvested by trypsinization at 48 hr post-transfection, and cell pellets were suspended in phosphate-buffered saline (PBS) buffer at 10^6^ cells/ml. The cell suspension was mixed 9:1 with 0.4% trypan blue solution and stained for 3 min. Then 10 μl of the stained cell suspension was drained and placed on a hemocytometer. The stained dead cells and unstained live cells were counted separately under the microscope. Every sample was counted three times. The percentage of viable cells was calculated by the following formula: cell viability = (N_total cells_—N_dead cells_)/ N_total cells_×100%.

### LINE-1 Retrotransposition Assay

The empty, the A3B-HA, or the A3DE-HA expression vector was co-transfected with 2 μg of LINE-1 plasmid separately into HEK293T cells in 12-well plates. The cells were selected by the addition of puromycin (final concentration, 5 μg/ml) at 48 hr post-transfection. GFP-positive cells were examined after another 48 hr by flow cytometry using FACSCalibur. Gating exclusions were set up based on background fluorescence of the cells transfected with the plasmid JM111; 20,000 single-cell events per sample were gated and analyzed using CellQuest Pro (v.5.2). The rest of the cells were collected to check the expression of the A3DE proteins after flow cytometry.

### LEAP Assay and RT-PCR

The LINE-1 construct pc-L1-1FH, containing the FLAG-HA-tagged ORF1, has been described [41]. It was co-transfected in the absence or presence of the A3DE-HA expression vector into HEK293T cells. After transfection for 2 days, LINE-1 ribonucleoprotein (RNP) complexes were isolated by ultracentrifugation through a sucrose cushion as previously described [42]. The LINE-1 RNP sample (2 μl) was added to each cDNA extension reaction (LEAP) using the 3’ RACE adaptor NV: 5’-GCGAGCACAGAATTAATACGACTCACTATAGGTTTTTTTTTTTTVN-3’ as primer. LINE-1 RNA was extracted from the LINE-1 RNP, treated with DNase I (Promega), and reverse-transcribed using the 3’ RACE adaptors NV as primer and MuLV RT, using the GoScript Reverse Transcription System (Promega). PCR was performed as previously described [42]. The relative amount of synthesized cDNA from both methods was detected by real-time PCR using the primers Linker (as part of the primer 3’ RACE adaptor NV), 5’-GCGAGCACAGAATTAATACGACT-3’ and L1-LEAP-R, 5’-GGGTTCGAAATCGATAAGCTTGGATCCAGAC-3’. FastStart Universal SYBR Green Master Mix (Roche) was used for qRT-PCR amplifications. The reactions were performed under the following conditions: 50°C for 2 min and 95°C for 10 min, then 40 cycles of 95°C for 15 s and 60°C for 1 min, followed by a dissociation protocol. Calculations were performed using the 2^-ΔΔCT^ method.

### Protein Extraction and Western Blotting

Cell pellets were lysed in RIPA buffer (50 mM Tris, pH7.4, with 150 mM NaCl, 1% NP40, 9 mM ethylenediamine tetra acetic acid (EDTA)) and boiled for 30 min in a 100°C water bath. The following antibodies were used to detect protein expression: anti-HA from Invitrogen (Carlsbad, CA), anti-Flag agarose gel from Sigma, anti-V5 and anti-EGFP from Invitrogen, and anti-tubulin from Abcam (Cambridge, MA). All antibodies were used according to the manufacturers’ protocols.

### Immunoprecipitation

HEK293T cells in T25 flasks were transfected with 6 μg of pc-L1-1FH and 2 μg of A3DE-V5 vector, then cultured for 48 hr. ORF1 was tagged with Flag and HA in the pc-L1-1FH plasmid [41]. For each IP reaction, 3x10^7^ cells were harvested in PBS and pelleted, and then 1.0 ml of the whole-cell extracts was prepared with lysis buffer (50 mM Tris, pH7.4, with 150 mM NaCl, 1mM EDTA, 1% Triton X-100, and complete Mini EDTA-free protease inhibitor cocktail (Roche)). Extracts were incubated with 40 μl of anti-FLAG M2 affinity gel (Sigma), rotating for 3 hr at room temperature. Then the affinity gel was washed five times with buffer A (150 mM NaCl/50mM Tris–HCl (pH 7.5)) and the protein was eluted with 100 mM glycine (pH 2.5). The eluates were analyzed by SDS-PAGE and immunoblotting with the appropriate antibodies.

### RT-PCR

In brief, RNA was isolated using the RNeasy Mini Kit (Qiagen, Valencia, CA, USA). Then 1 μg RNA per reaction was treated with DNase I by incubation in 10 μl of diethyl pyrocarbonate (DEPC)-treated water with 10x RQ1 RNase-free DNase buffer, 1 μl of RQ1 RNase-free DNase (Promega), and 4U of RNase inhibitor (Promega) for 30 min at 37°C. The DNase was inactivated by addition of 1 μl of RQ1 DNase stop solution and incubation at 65°C for 10 min. Reverse transcription was performed using the GoScript Reverse Transcription System (Promega). 2x EasyTaq PCR SuperMix (Transgen) was used for PCR amplifications. The same amounts of cDNA template (2 μl) were subject to PCR with primers 5’- AGGAAATACAGAGAACGCCACAA-3’ and 5’- GCTGGATATGAAATTCTGGGTTGA -3’ that amplify ORF1 mRNA. GAPDH was used to normalize the amount of ORF1 mRNA. GAPDH mRNA was amplified with primers 5’- GCAAATTCCATGGCACCGT -3’ and 5’- TCGCCCCACTTGATTTTGG -3’. The reactions were performed under the following conditions: 94°C for 5 min, and 35 cycles of 94°C for 30 s, 57°C for 30s and 72°C for 30s, then 72°C for 10min. The PCR products were separated in 2% agarose gels and visualized with ethidium bromide staining.

### Immunofluorescence Microscopy

Cells were grown on glass coverslips before transfection with A3DE and L1-1FH. Forty-eight hours after transfection, cells were fixed with 100% methanol for 15 min at -20°C. Cells were blocked with 5% of goat serum for 60 min at room temperature and then incubated with primary antibody, anti-V5 (1:100 dilution), anti-HA(1:200 dilution), 4°C overnight. Alex Fluor 488-labeled goat anti-rabbit antibody (1:100 dilution), Alexa Fluor 594-labeled goat anti-mouse antibody (1:100 dilution) were used as secondary antibodies. DAPI (1:5000 dilution) was used to stain the nucleus. Immunofluorescence images were acquired at room temperature using Olympus IX73 fluorescence microscope. Data analysis and images processing were performed using cellSens software.

## Results

### APOBEC3DE inhibits human LINE-1 retrotransposition

**Fig 1:**
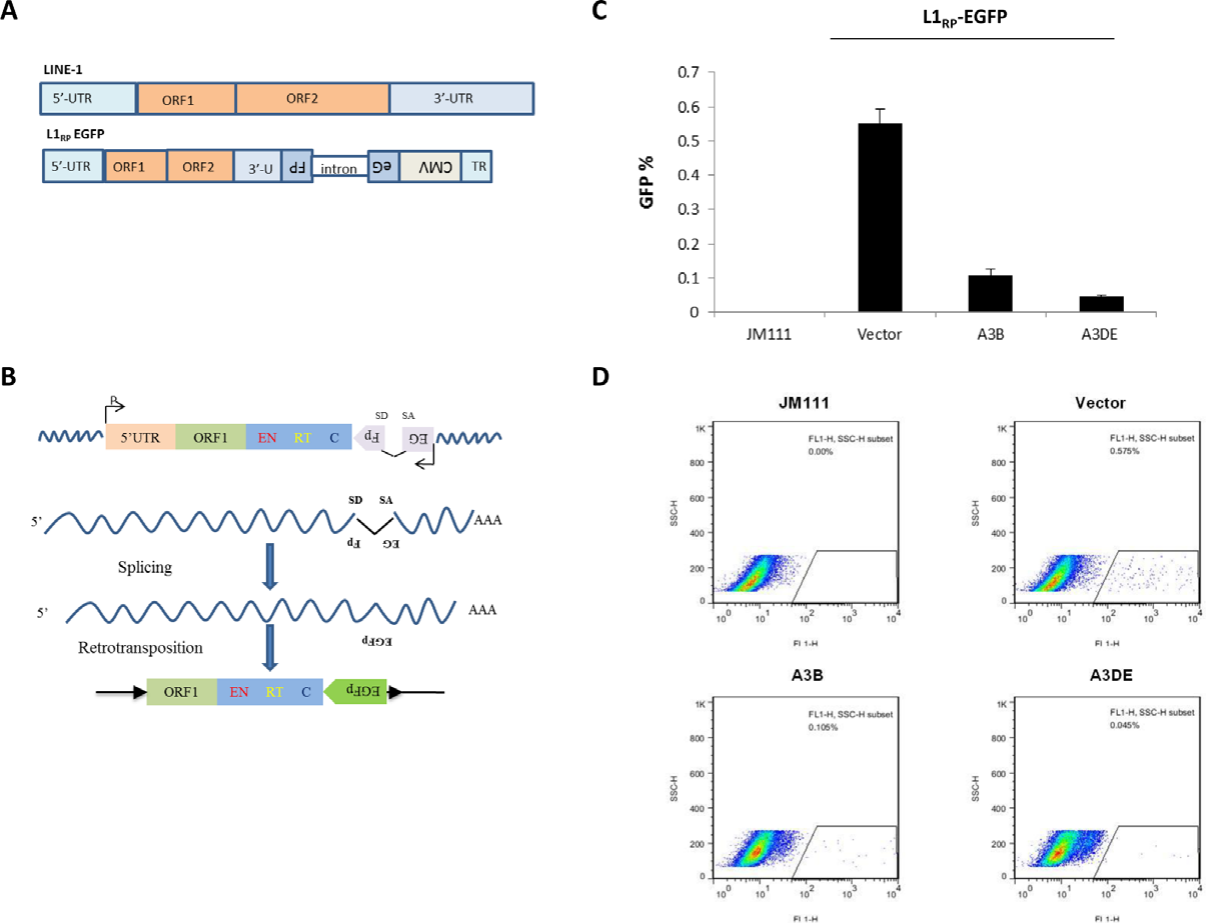
APOBEC3B and APOBEC3DE inhibit human LINE-1 retrotransposition. (A) Schematic diagram of the full-length LINE-1 element and pc-L1RP EGFP plasmids. Canonical L1 contains a 5’ untranslated region (5’UTR), two open reading frames (ORF1 and ORF2), and a 3’UTR, including a polyadenylation signal (AATAAA) and polyA tail (A)n. pc-L1RP EGFP contains an antisense cassette of EGFP that is interrupted by an intron. The LINE-1 5’UTR promoter initiates ORF1 and ORF2 expression, and EGFP is under the control of the CMV promoter in trans. (B) Retrotransposition assay of LINE-1. EGFP can only be expressed when LINE-1 is transposed into the genome with the intron removed during RNA splicing. (C) Inhibitory effect of A3B and A3DE on LINE-1 mobility. The A3B- or A3DE-expressing vector was co-transfected with L1RP EGFP, and flow cytometry was used to detect the EGFP-positive cells. The pcDNA3.1 vector was used as the control. The bar charts represent the results from three independent experiments; error bars indicate the S.D. of three replicates within one experiment. (D) Representative flow cytometry dot diagrams for A3B and A3DE in LINE-1 restriction. The A3B-HA- or A3DE-HA-expressing vector was co-transfected with L1RP EGFP into HEK293T cells. JM111 transfected cells, as a negative control, were used to gate the EGFP-positive cells. The percentage of EGFP-positive events is shown in the upper right corner of each panel.

To address the effects of A3B and A3DE on LINE-1 retrotransposition, we used a well-established EGFP reporter system in HEK293T cells [34, 40, 43, 44] to evaluate LINE-1 retrotransposition in the presence or absence of A3B or A3DE (Fig 1A and 1B). EGFP was expressed only after the LINE-1 transcript was spliced and reverse-transcribed, its cDNA was inserted into the host genome, and the EGFP reporter gene was transcribed under the control of its own CMV promoter (Fig 1B). Construct 99 PUR JM111 EGFP (JM111), which contains two missense mutations in ORF1, was used as a negative control for retrotransposition. The pL1RP EGFP construct was transfected into HEK293T cells with or without the A3B or A3DE expression plasmids. After 48 hr, the cells were selected by puromycin, and the number of GFP-positive cells was examined after additional 48 hr by flow cytometry. Gating exclusions were set up based on the background fluorescence of the cells transfected with the plasmid JM111, as previously described [34]. We observed that both A3B and A3DE had a significant inhibitory effect (up to ~80%, *p*<0.05) on LINE-1 retrotransposition (Fig 1C). Representative flow cytometry data are shown in Fig 1D.

**Fig 2:**
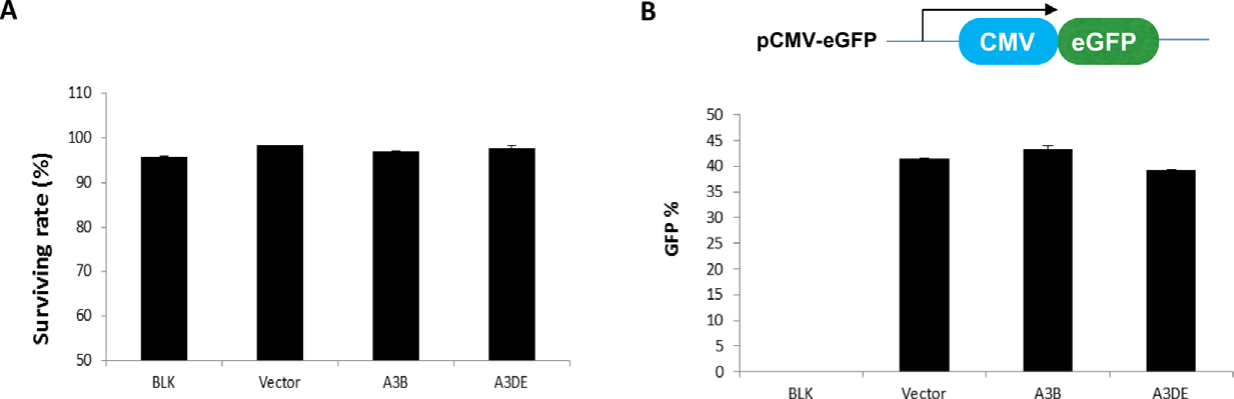
Neither APOBEC3B nor APOBEC3DE affects EGFP expression, and both do not cause cytotoxicity in HEK293T cells. (A) No cytotoxicity was observed in HEK293T cells transfected with L1RP-EGFP together with A3B or ADE at 48 hr post-transfection, when compared to the control. Cells were stained with trypan blue, and the percentage of living cells was calculated as the % survival. (B) The empty vector, A3B and A3DE was co-transfected with, pcDNA3.1-EGFP plasmid in HEK293T cells and EGFP-positive cells were detected by flow cytometry at 48 hr post-transfection.

It was important to exclude any possible toxic effects of the A3B or A3DE protein that would bias the results. Trypan blue staining was used to assess cell viability and cytotoxicity in HEK293T cells transfected with the A3B or A3DE expression vector plus L1RP-EGFP. Expression of either the A3B or A3DE protein caused no detectable cytotoxicity in HEK293T cells (Fig 2A).

The LINE-1 retrotransposition assay relies on the detection of EGFP driven by the CMV promoter. By using flow cytometry, we demonstrated that the expression of the A3B and A3DE proteins did not affect the positive EGFP number when co-transfected with pcDNA-EGFP plasmids (Fig 2B). Hence, we had established an optimal system for studying A3-mediated LINE-1 restriction.

### APOBEC3DE interacts with ORF1p in a RNA-dependent manner

**Fig 3:**
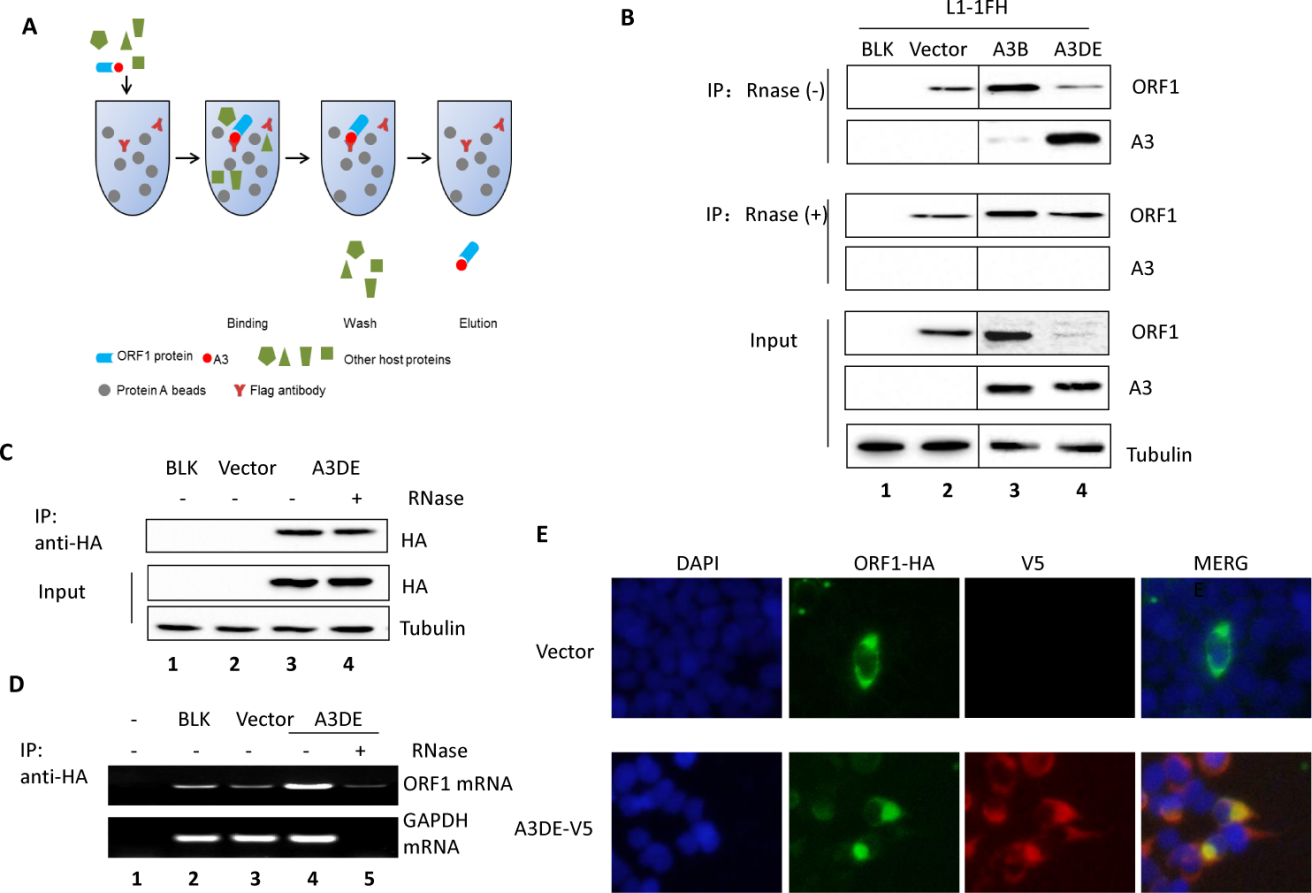
APOBEC3DE interacts with ORF1p in a RNA-dependent manner. (A) Diagram of the co-IP assay. Cleared whole-cell lysate was used for these assays. Protein antigens were pulled down by specific antibodies. The antibodies were coupled to solid substrates. (B) A3B or A3DE interacts with ORF1p, and RNase disrupts the binding of A3F to ORF1p. The pc-L1-1FH plasmid was co-transfected with A3B-V5 or A3DE-V5 or the control vector into HEK293T cells. The cleared cell lysate was split into two halves, and RNase A was added to one half (final concentration, 50 μg/ml); both samples were then incubated with Flag-tagged beads. Western blotting was performed to identify the input, RNase A-treated, and untreated IP products. (C, D) IP of A3DE for endogenous L1 RNP. A3DE-HA was transfected into HEK293T cells and HA beads were used to pull down A3DE. IP assay was conducted at 48 hours post-transfection. IP product was aliquoted, one for Western Blotting and the other one for RNA extraction and L1 mRNA detection. (E) Immunofluorescence staining of A3DE and ORF1p in HEK293T cells.

We next examined whether the human A3B or A3DE could target LINE-1 RNP and affect LINE-1 activity. ORF1 shares little homology with known proteins, and LINE-1 RNA is wrapped around by the ORF1p trimer [45]. Both ORF1p and ORF2p are components of LINE-1 RNP and are required for retrotransposition during target site-primed reverse transcription (TPRT) [46]. Therefore, we measured the interaction of the A3B and A3DE proteins with LINE-1 ORF1p in a co-immunoprecipitation assay (Fig 3A). Immunoprecipitation (IP) was performed using Flag epitope-tagged ORF1p (pc-L1-1FH) co-expressed with A3B or A3DE. Using an anti-FLAG-agarose purification from transfected HEK293T cells, we demonstrated that A3DE interacted with ORF1p (Fig 3B). Furthermore, this interaction could be disrupted by RNase treatment (Fig 3B), indicating that A3DE proteins target LINE-1 RNP by interacting with ORF1p in a RNA-dependent manner. Although A3B had potent activity against LINE-1 retrotransposition, its interaction with ORF1p (Fig 3B, lane 3) was less efficient than that of A3DE (Fig 3B, lane 4). The weak interaction between A3B and ORF1p disappeared after RNase treatment (Fig 3B, lane 3) consistent with a previous report that A3B and ORF1p form a RNA-dependent complex [47]. Moreover, we examined whether A3DE could pull down the endogenous L1 mRNA. A3DE-HA transfected HEK293T cells were collected, lysed, and immunoprecipitated with anti-HA antibody (Fig 3C). The L1 mRNA in the eluted samples was detected by real-time PCR. Our data showed that A3DE binds with L1 mRNA of L1 RNP in a RNA dependent manner (Fig 3D, lane 4 and lane 5). Subsequently, we further detected the subcellular localization of A3DE and ORF1p in HEK293T cells (Fig 3E) and found that A3DE was co-localized with ORF1p in the cytoplasm. All these results support that A3DE interacts with L1 RNP.

### APOBEC3DE inhibits ORF2p-mediated reverse transcription of LINE-1 RNP

**Fig 4:**
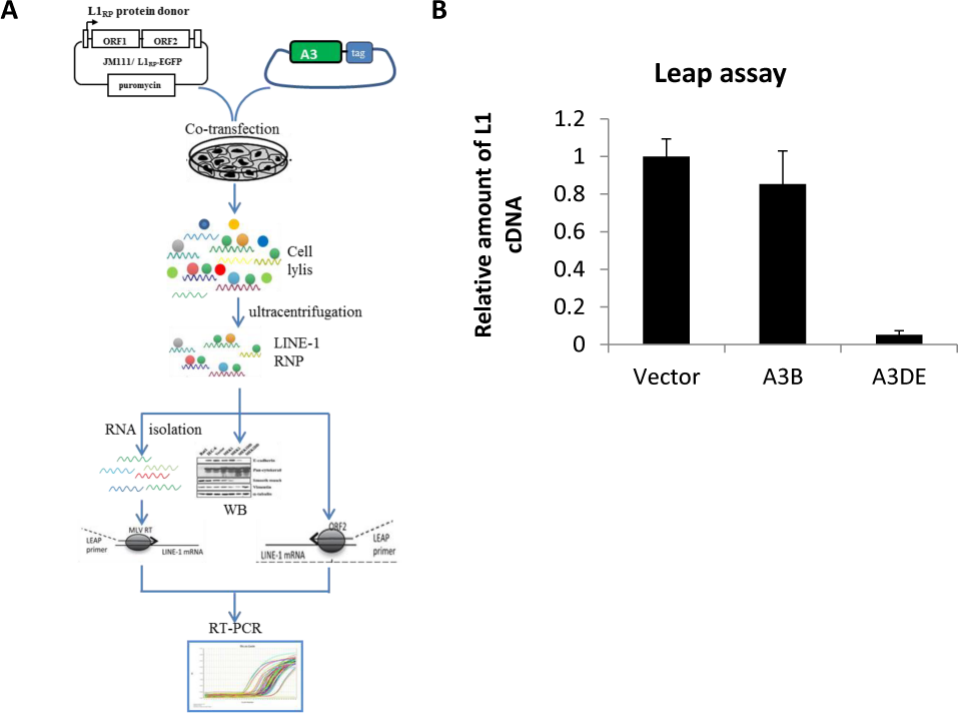
APOBEC3DE targets LINE RNP particles and inhibits ORF2p function. (A) Flow chart for the LEAP assay. pc-L1-1FH was co-transfected with A3B or A3DE or empty vector into HEK293T cells. The purified LINE-1 RNPs from pc-L1-1FH were used for three assays: LEAP, MLV RT-PCR, and western blotting. The LEAP primer was used to target LINE-1 mRNA through the designed linker region. Real-time PCR was used to amplify the L1 cDNA. The ratio of LEAP to MLV is expressed as the relative L1 cDNA amount. (B) Quantitative real-time PCR analysis of LEAP and MLV RT products. A3B or A3DE was co-transfected with the pc-L1-1FH plasmid into HEK293T cells, and cell lysates were prepared at 48 hr post-transfection. The relative amounts of synthesized cDNA from LEAP and MLV-RT were detected with the Linker primer and the L1-LEAP-R primer (as indicated in the Methods) by real-time PCR. A3DE reduced the reverse transcription activity of the L1 ORF2p when compared to the vector control.

LINE-1 replication requires reverse transcription of its own RNA genome using ORF2p. To further understand the mechanism of A3-mediated LINE-1 inhibition, we investigated whether A3B or A3DE could affect LINE-1 ORF2p’s involvement in LINE-1 RNP transcription. To detect the TPRT activity, a LEAP reverse transcriptase assay (Fig 4A), established by Kulpa et al., was used to evaluate the LINE-1 RT activity of ORF2p in LINE-1 RNP [42]. LINE-1 RNPs from an ORF1-tagged LINE-1 construct [41] were isolated from transfected HEK293T cells in the absence or presence of A3B- or A3DE-expressing vector. In our study, A3DE showed potent inhibition of LINE-1 reverse transcriptase activity (Fig 4B). The amount of LINE-1 complementary DNA synthesized by LINE-1 reverse transcriptase was reduced by ~75% (*p*<0.005). In sharp contrast to A3DE, expression of A3B had only a weak inhibitory effect on LINE-1 reverse transcriptase activity (Fig 4B). Thus, our studies revealed that A3DE could associate with LINE-1 RNP and affect ORF2p’s reverse transcriptase activity.

### APOBEC3DE restrict LINE-1 retrotransposition through a DNA deamination-independent mechanism

**Fig 5:**
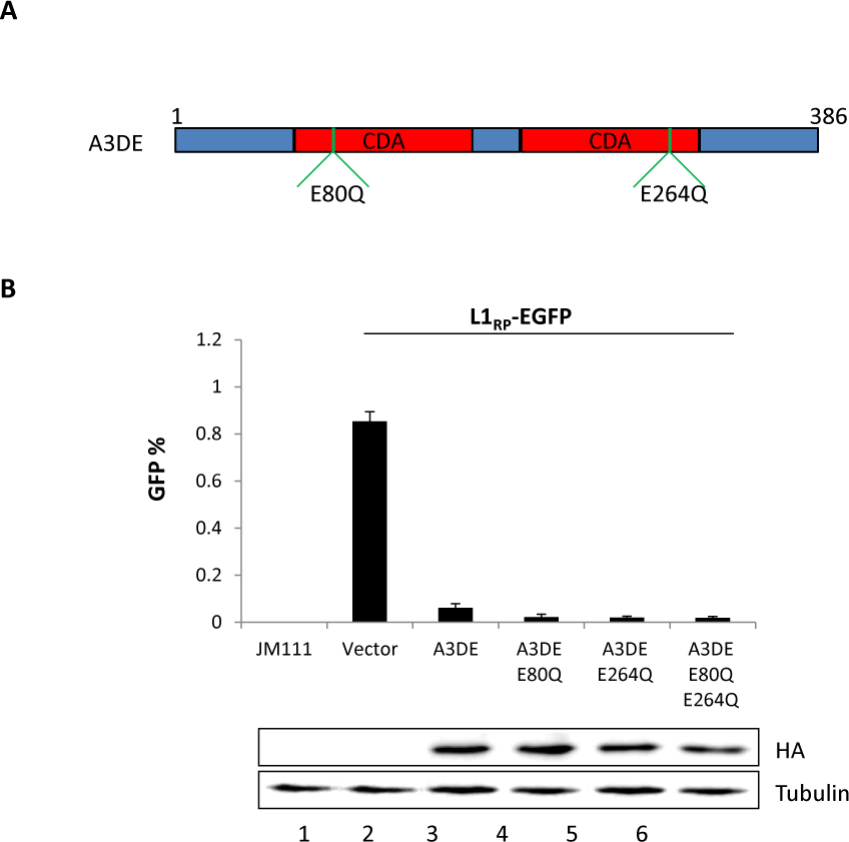
APOBEC3DE CDA mutations maintained the inhibitory ability on LINE-1 retrotransposition. (A) Diagram of A3DE cytidine deaminase domains. (B) The effects of A3DE mutations on LINE-1 restriction by flow cytometry. The effects of the WT and mutant A3DE on LINE-1 restriction were compared under the similar A3DE protein expression levels.

A3DE cytosine deaminase (CDA) domain is crucial for anti-HIV-1 infection. A3DE cytidine deaminase domain mutations, A3DE-E80Q and A3DE-E264Q lost the ability antagonize HIV-1 dVif [48]. In this study, we observed that A3DE-E80Q, A3DE-E264Q and A3DE-E80Q/E264Q keep the same potent inhibitory effects on LINE-1 as A3DE wild type (Fig 5). It had been reported that A3B, A3C and A3F have varied abilities inhibiting LINE-1 through CDA-independent pathway [35, 36]. We also examined A3B CDA mutations, A3B-W228L/ D316N on LINE1 restriction and obtained a similar result as published (Data not shown). Herein, we demonstrated that A3DE, another A3 family member, could also inhibit LINE-1 mobility through CDA-independent pathway.

## Discussion

Human A3 proteins, as well as polymorphic variants of certain A3 proteins, show differences in their ability to restrict LINE-1 retrotransposition. In general, A3A has been considered to be the most potent inhibitor of LINE-1 retrotransposition. In contrast, A3G has been shown to demonstrate very little or no activity against LINE-1 retrotransposition. Previous studies have indicated that human polymorphism in A3H has resulted in altered capacities for LINE-1 restriction. A3H Hap-II has a strong ability to restrict LINE-1, while A3H Hap-III and Hap-IV have lost this function. Results generated from the present study indicate that human A3DE is another potent cytidine deaminase with the ability to restrict LINE-1. A3DE is expressed in embryonic stem cells, and both human A3B and A3DE may play important roles in regulating the mobilization of endogenous retroelements *in vivo* [21, 24, 30].

The mechanisms of LINE-1 inhibition by various A3 proteins have not been fully characterized. A3A can inhibit LINE-1 retrotransposition, which requires its deaminase activity. It has been proposed that A3A induces cytidine deamination of transiently exposed LINE-1 single-strand DNA produced during the process of LINE-1 reverse transcription/integration [43]. In this study, we have detected a physical interaction between LINE-1 ORF1p and A3DE by co-immunoprecipitation experiments. The interaction between ORF1p and A3DE was sensitive to RNase treatment, indicating the involvement of RNA. In contrast, A3B had a much weaker ability to interact with LINE-1 ORF1p when compared to A3DE (Fig 3), despite the fact that both proteins showed evidence of strong inhibitory effects on LINE-1 retrotransposition (Fig 1). The nature of the RNA molecule that mediates the ORF1p-A3DE interaction is not clear. LINE-1 RNA is a likely candidate. Alternatively, it has also been reported that 7SL RNA is associated with LINE-1 RNPs [49]. We have previously observed an association of 7SL RNA with A3 proteins [17, 50]. The involvement of 7SL RNA in mediating the interaction between ORF1p and A3DE is another possibility.

Several studies revealed that a cytoplasmic granule activation pathway is involved in LINE-1 restriction by cellular factors, such as SAMHD1, MOV10 and ZAP [32, 33, 41]. Here we also discovered the co-localization of A3DE with L1 ORF1p in cytoplasmic granules. Further study is needed to clarify which kind of cellular granules play a role in A3DE inhibition of LINE-1 mobility.

We investigated whether A3DE can interfere with LINE-1 RT activity, since A3DE interacts with ORF1p and associates with LINE-1 RNPs. We observed an ~80% A3DE-associated inhibition of LINE-1 RT activity using purified LINE-1 RNPs in the LEAP assay (Fig 4). This magnitude of inhibition mirrors that of LINE-1 inhibition by A3DE in the cell culture assay. The impairment of LINE-1 RT activity by A3DE is also similar to the reported effect of deaminase-defective A3G and A3F on the RT activity of HIV-1Δvif virus in newly infected target cells [51]. A similar but less potent inhibition of LINE-1 RT activity has also been reported for A3C [35]. We have observed a much weaker inhibitory effect on LINE-1 RT activity for A3B than for A3DE (Fig 4). A3DE inhibits LINE-1 retrotransposition by using DNA deaminase-independent mechanism, which is distinct from the one used by A3A. In any case, it is apparent that human A3 proteins have developed different mechanisms for LINE-1 regulation.

## References

[1] Chiu YL, Witkowska HE, Hall SC, Santiago M, Soros VB, Esnault C, et al High-molecular-mass APOBEC3G complexes restrict Alu retrotransposition. Proc Natl Acad Sci U S A. [Journal Article; Research Support, N.I.H., Extramural; Research Support, Non-U.S. Gov't]. 2006 2006-10-17;103(42):15588–93. 17030807 10.1073/pnas.0604524103 PMC1592537

[2] Conticello SG. The AID/APOBEC family of nucleic acid mutators. GENOME BIOL. [Journal Article; Research Support, Non-U.S. Gov't; Review]. 2008 2008-1-20;9(6):229 10.1186/gb-2008-9-6-229 18598372 PMC2481415

[3] Goila-Gaur R, Strebel K. HIV-1 Vif, APOBEC, and intrinsic immunity. RETROVIROLOGY. [Journal Article; Research Support, N.I.H., Intramural; Review]. 2008 2008-1-20;5:51 10.1186/1742-4690-5-51 18577210 PMC2443170

[4] Kinomoto M, Kanno T, Shimura M, Ishizaka Y, Kojima A, Kurata T, et al All APOBEC3 family proteins differentially inhibit LINE-1 retrotransposition. NUCLEIC ACIDS RES. [Journal Article; Research Support, Non-U.S. Gov't]. 2007 2007-1-20;35(9):2955–64. 17439959 10.1093/nar/gkm181 PMC1888823

[5] Malim MH. Natural resistance to HIV infection: The Vif-APOBEC interaction. C R Biol. [Journal Article; Research Support, Non-U.S. Gov't]. 2006 2006-11-01;329(11):871–5. 17067930 10.1016/j.crvi.2006.01.012

[6] Muckenfuss H, Hamdorf M, Held U, Perkovic M, Lower J, Cichutek K, et al APOBEC3 proteins inhibit human LINE-1 retrotransposition. J BIOL CHEM. [Journal Article; Research Support, Non-U.S. Gov't]. 2006 2006-8-04;281(31):22161–72. 16735504 10.1074/jbc.M601716200

[7] Harris RS, Liddament MT. Retroviral restriction by APOBEC proteins. NAT REV IMMUNOL. [Journal Article; Research Support, Non-U.S. Gov't; Review]. 2004 2004-11-01;4(11):868–77. 15516966 10.1038/nri1489

[8] Conticello SG, Harris RS, Neuberger MS. The Vif protein of HIV triggers degradation of the human antiretroviral DNA deaminase APOBEC3G. CURR BIOL. [Journal Article; Research Support, Non-U.S. Gov't]. 2003 2003-11-11;13(22):2009–13. 14614829 10.1016/j.cub.2003.10.034

[9] Cullen BR. HIV-1 Vif: counteracting innate antiretroviral defenses. MOL THER. [Journal Article]. 2003 2003-10-01;8(4):525–7. 14565218 10.1016/j.ymthe.2003.08.010

[10] Larue RS, Lengyel J, Jonsson SR, Andresdottir V, Harris RS. Lentiviral Vif degrades the APOBEC3Z3/APOBEC3H protein of its mammalian host and is capable of cross-species activity. J VIROL. [Journal Article; Research Support, N.I.H., Extramural; Research Support, Non-U.S. Gov't]. 2010 2010-8-01;84(16):8193–201. 10.1128/JVI.00685-10 20519393 PMC2916508

[11] Marin M, Rose KM, Kozak SL, Kabat D. HIV-1 Vif protein binds the editing enzyme APOBEC3G and induces its degradation. NAT MED. [Journal Article; Research Support, Non-U.S. Gov't; Research Support, U.S. Gov't, P.H.S.]. 2003 2003-11-01;9(11):1398–403. 14528301 10.1038/nm946

[12] Sheehy AM, Gaddis NC, Malim MH. The antiretroviral enzyme APOBEC3G is degraded by the proteasome in response to HIV-1 Vif. NAT MED. [Journal Article; Research Support, Non-U.S. Gov't; Research Support, U.S. Gov't, Non-P.H.S.]. 2003 2003-11-01;9(11):1404–7. 14528300 10.1038/nm945

[13] Zhang W, Du J, Evans SL, Yu Y, Yu XF. T-cell differentiation factor CBF-beta regulates HIV-1 Vif-mediated evasion of host restriction. NATURE. [Journal Article; Research Support, N.I.H., Extramural; Research Support, Non-U.S. Gov't]. 2012 2012-1-19;481(7381):376–9. 10.1038/nature10718 22190036

[14] Zhen A, Wang T, Zhao K, Xiong Y, Yu XF. A single amino acid difference in human APOBEC3H variants determines HIV-1 Vif sensitivity. J VIROL. [Journal Article; Research Support, N.I.H., Extramural]. 2010 2010-2-01;84(4):1902–11. 10.1128/JVI.01509-09 19939923 PMC2812409

[15] Alce TM, Popik W. APOBEC3G is incorporated into virus-like particles by a direct interaction with HIV-1 Gag nucleocapsid protein. J BIOL CHEM. [Journal Article; Research Support, U.S. Gov't, P.H.S.]. 2004 2004-8-13;279(33):34083–6. 15215254 10.1074/jbc.C400235200

[16] Schafer A, Bogerd HP, Cullen BR. Specific packaging of APOBEC3G into HIV-1 virions is mediated by the nucleocapsid domain of the gag polyprotein precursor. VIROLOGY. [Journal Article; Research Support, Non-U.S. Gov't; Research Support, U.S. Gov't, P.H.S.]. 2004 2004-10-25;328(2):163–8. 15464836 10.1016/j.virol.2004.08.006

[17] Wang T, Tian C, Zhang W, Luo K, Sarkis PT, Yu L, et al 7SL RNA mediates virion packaging of the antiviral cytidine deaminase APOBEC3 G. J VIROL. [Journal Article; Research Support, N.I.H., Extramural; Research Support, Non-U.S. Gov't]. 2007 2007-12-01;81(23):13112–24. 10.1128/JVI.00892-07 PMC2169093 17881443

[18] Zennou V, Perez-Caballero D, Gottlinger H, Bieniasz PD. APOBEC3G incorporation into human immunodeficiency virus type 1 particles. J VIROL. [Journal Article; Research Support, Non-U.S. Gov't; Research Support, U.S. Gov't, P.H.S.]. 2004 2004-11-01;78(21):12058–61. 15479846 10.1128/JVI.78.21.12058-12061.2004 PMC523273

[19] Cen S, Guo F, Niu M, Saadatmand J, Deflassieux J, Kleiman L. The interaction between HIV-1 Gag and APOBEC3G. J BIOL CHEM. [Journal Article; Research Support, Non-U.S. Gov't]. 2004 2004-8-06;279(32):33177–84. 15159405 10.1074/jbc.M402062200

[20] Luo K, Liu B, Xiao Z, Yu Y, Yu X, Gorelick R, et al Amino-terminal region of the human immunodeficiency virus type 1 nucleocapsid is required for human APOBEC3G packaging. J VIROL. [Journal Article]. 2004 2004-11-01;78(21):11841–52. 15479826 10.1128/JVI.78.21.11841-11852.2004 PMC523292

[21] Schumann GG, Gogvadze EV, Osanai-Futahashi M, Kuroki A, Munk C, Fujiwara H, et al Unique functions of repetitive transcriptomes. Int Rev Cell Mol Biol. [Journal Article; Research Support, Non-U.S. Gov't; Review]. 2010 2010-1-20;285:115–88. 10.1016/B978-0-12-381047-2.00003-7 21035099

[22] Beck CR, Garcia-Perez JL, Badge RM, Moran JV. LINE-1 elements in structural variation and disease. Annu Rev Genomics Hum Genet. [Journal Article; Research Support, N.I.H., Extramural; Research Support, Non-U.S. Gov't; Review]. 2011 2011-1-20;12:187–215. 10.1146/annurev-genom-082509-141802 21801021 PMC4124830

[23] Brouha B, Schustak J, Badge RM, Lutz-Prigge S, Farley AH, Moran JV, et al Hot L1s account for the bulk of retrotransposition in the human population. Proc Natl Acad Sci U S A. [Journal Article; Research Support, Non-U.S. Gov't; Research Support, U.S. Gov't, P.H.S.]. 2003 2003-4-29;100(9):5280–5. 12682288 10.1073/pnas.0831042100 PMC154336

[24] Hancks DC, Kazazian HJ. Active human retrotransposons: variation and disease. CURR OPIN GENET DEV. [Journal Article; Research Support, N.I.H., Extramural; Review]. 2012 2012-6-01;22(3):191–203. 10.1016/j.gde.2012.02.006 22406018 PMC3376660

[25] Lander ES, Linton LM, Birren B, Nusbaum C, Zody MC, Baldwin J, et al Initial sequencing and analysis of the human genome. NATURE. [Journal Article; Research Support, Non-U.S. Gov't; Research Support, U.S. Gov't, Non-P.H.S.; Research Support, U.S. Gov't, P.H.S.]. 2001 2001-2-15;409(6822):860–921. 11237011 10.1038/35057062

[26] Ostertag EM, Goodier JL, Zhang Y, Kazazian HJ. SVA elements are nonautonomous retrotransposons that cause disease in humans. AM J HUM GENET. [Comparative Study; Journal Article; Research Support, Non-U.S. Gov't; Research Support, U.S. Gov't, P.H.S.]. 2003 2003-12-01;73(6):1444–51. 14628287 10.1086/380207 PMC1180407

[27] Wang H, Xing J, Grover D, Hedges DJ, Han K, Walker JA, et al SVA elements: a hominid-specific retroposon family. J MOL BIOL. [Journal Article; Research Support, N.I.H., Extramural; Research Support, Non-U.S. Gov't; Research Support, U.S. Gov't, Non-P.H.S.]. 2005 2005-12-09;354(4):994–1007. 16288912 10.1016/j.jmb.2005.09.085

[28] Dombroski BA, Mathias SL, Nanthakumar E, Scott AF, Kazazian HJ. Isolation of an active human transposable element. SCIENCE. [Comparative Study; Journal Article; Research Support, Non-U.S. Gov't; Research Support, U.S. Gov't, P.H.S.]. 1991 1991-12-20;254(5039):1805–8. 1662412 10.1126/science.1662412

[29] Feng Q, Moran JV, Kazazian HJ, Boeke JD. E15—Human L1 retrotransposon encodes a conserved endonuclease required for retrotransposition. CELL. [Journal Article; Research Support, Non-U.S. Gov't; Research Support, U.S. Gov't, P.H.S.]. 1996 1996-11-29;87(5):905–16. 8945517 10.1016/s0092-8674(00)81997-2

[30] Goodier JL, Kazazian HJ. Retrotransposons revisited: the restraint and rehabilitation of parasites. CELL. [Journal Article; Research Support, N.I.H., Extramural; Research Support, U.S. Gov't, Non-P.H.S.; Review]. 2008 2008-10-03;135(1):23–35. 10.1016/j.cell.2008.09.022 18854152

[31] Li X, Zhang J, Jia R, Cheng V, Xu X, Qiao W, et al The MOV10 helicase inhibits LINE-1 mobility. J BIOL CHEM. [Journal Article; Research Support, Non-U.S. Gov't]. 2013 2013-7-19;288(29):21148–60. 10.1074/jbc.M113.465856 23754279 PMC3774381

[32] Hu S, Li J, Xu F, Mei S, Le Duff Y, Yin L, et al SAMHD1 Inhibits LINE-1 Retrotransposition by Promoting Stress Granule Formation. PLOS GENET. [Journal Article; Research Support, Non-U.S. Gov't]. 2015 2015-7-01;11(7):e1005367 10.1371/journal.pgen.1005367 26134849 PMC4489885

[33] Moldovan JB, Moran JV. The Zinc-Finger Antiviral Protein ZAP Inhibits LINE and Alu Retrotransposition. PLOS GENET. [Journal Article; Research Support, N.I.H., Extramural; Research Support, Non-U.S. Gov't]. 2015 2015-5-01;11(5):e1005121 10.1371/journal.pgen.1005121 25951186 PMC4423928

[34] Zhao K, Du J, Han X, Goodier JL, Li P, Zhou X, et al Modulation of LINE-1 and Alu/SVA retrotransposition by Aicardi-Goutieres syndrome-related SAMHD1. CELL REP. [Journal Article; Research Support, N.I.H., Extramural; Research Support, Non-U.S. Gov't]. 2013 2013-9-26;4(6):1108–15. 10.1016/j.celrep.2013.08.019 24035396 PMC3988314

[35] Horn AV, Klawitter S, Held U, Berger A, Vasudevan AA, Bock A, et al Human LINE-1 restriction by APOBEC3C is deaminase independent and mediated by an ORF1p interaction that affects LINE reverse transcriptase activity. NUCLEIC ACIDS RES. [Journal Article; Research Support, Non-U.S. Gov't]. 2014 2014-1-01;42(1):396–416. 10.1093/nar/gkt898 24101588 PMC3874205

[36] Stenglein MD, Harris RS. APOBEC3B and APOBEC3F inhibit L1 retrotransposition by a DNA deamination-independent mechanism. J BIOL CHEM. [Journal Article; Research Support, N.I.H., Extramural; Research Support, Non-U.S. Gov't]. 2006 2006-6-23;281(25):16837–41. 16648136 10.1074/jbc.M602367200

[37] Gallois-Montbrun S, Holmes RK, Swanson CM, Fernandez-Ocana M, Byers HL, Ward MA, et al Comparison of cellular ribonucleoprotein complexes associated with the APOBEC3F and APOBEC3G antiviral proteins. J VIROL. [Comparative Study; Journal Article; Research Support, Non-U.S. Gov't]. 2008 2008-6-01;82(11):5636–42. 10.1128/JVI.00287-08 18367521 PMC2395208

[38] Chen H, Lilley CE, Yu Q, Lee DV, Chou J, Narvaiza I, et al APOBEC3A is a potent inhibitor of adeno-associated virus and retrotransposons. CURR BIOL. [Journal Article; Research Support, N.I.H., Extramural; Research Support, Non-U.S. Gov't]. 2006 2006-3-07;16(5):480–5. 16527742 10.1016/j.cub.2006.01.031

[39] Bogerd HP, Wiegand HL, Hulme AE, Garcia-Perez JL, O'Shea KS, Moran JV, et al Cellular inhibitors of long interspersed element 1 and Alu retrotransposition. Proc Natl Acad Sci U S A. [Journal Article; Research Support, N.I.H., Extramural; Research Support, Non-U.S. Gov't]. 2006 2006-6-06;103(23):8780–5. 16728505 10.1073/pnas.0603313103 PMC1482655

[40] Ostertag EM, Prak ET, DeBerardinis RJ, Moran JV, Kazazian HJ. Determination of L1 retrotransposition kinetics in cultured cells. NUCLEIC ACIDS RES. [Journal Article; Research Support, Non-U.S. Gov't; Research Support, U.S. Gov't, P.H.S.]. 2000 2000-3-15;28(6):1418–23. 10684937 10.1093/nar/28.6.1418 PMC111040

[41] Goodier JL, Cheung LE, Kazazian HJ. MOV10 RNA helicase is a potent inhibitor of retrotransposition in cells. PLOS GENET. [Journal Article; Research Support, N.I.H., Extramural]. 2012 2012-1-20;8(10):e1002941 10.1371/journal.pgen.1002941 23093941 PMC3475670

[42] Kulpa DA, Moran JV. Cis-preferential LINE-1 reverse transcriptase activity in ribonucleoprotein particles. NAT STRUCT MOL BIOL. [Journal Article; Research Support, N.I.H., Extramural; Research Support, Non-U.S. Gov't]. 2006 2006-7-01;13(7):655–60. 16783376 10.1038/nsmb1107

[43] Moran JV, Holmes SE, Naas TP, DeBerardinis RJ, Boeke JD, Kazazian HJ. High frequency retrotransposition in cultured mammalian cells. CELL. [Journal Article; Research Support, Non-U.S. Gov't; Research Support, U.S. Gov't, P.H.S.]. 1996 1996-11-29;87(5):917–27. 8945518 10.1016/s0092-8674(00)81998-4

[44] Niewiadomska AM, Tian C, Tan L, Wang T, Sarkis PT, Yu XF. Differential inhibition of long interspersed element 1 by APOBEC3 does not correlate with high-molecular-mass-complex formation or P-body association. J VIROL. [Journal Article; Research Support, N.I.H., Extramural; Research Support, Non-U.S. Gov't]. 2007 2007-9-01;81(17):9577–83. 17582006 10.1128/JVI.02800-06 PMC1951403

[45] Khazina E, Truffault V, Buttner R, Schmidt S, Coles M, Weichenrieder O. Trimeric structure and flexibility of the L1ORF1 protein in human L1 retrotransposition. NAT STRUCT MOL BIOL. [Journal Article; Research Support, Non-U.S. Gov't]. 2011 2011-9-01;18(9):1006–14. 10.1038/nsmb.2097 21822284

[46] Luan DD, Korman MH, Jakubczak JL, Eickbush TH. Reverse transcription of R2Bm RNA is primed by a nick at the chromosomal target site: a mechanism for non-LTR retrotransposition. CELL. [Journal Article; Research Support, Non-U.S. Gov't; Research Support, U.S. Gov't, P.H.S.]. 1993 1993-2-26;72(4):595–605. 7679954 10.1016/0092-8674(93)90078-5

[47] Lovsin N, Peterlin BM. APOBEC3 proteins inhibit LINE-1 retrotransposition in the absence of ORF1p binding. Ann N Y Acad Sci. [Journal Article; Research Support, N.I.H., Extramural; Research Support, Non-U.S. Gov't]. 2009 2009-10-01;1178:268–75. 10.1111/j.1749-6632.2009.05006.x 19845642 PMC4475406

[48] Duggal NK, Malik HS, Emerman M. The breadth of antiviral activity of Apobec3DE in chimpanzees has been driven by positive selection. J VIROL. [Journal Article; Research Support, N.I.H., Extramural; Research Support, Non-U.S. Gov't; Research Support, U.S. Gov't, Non-P.H.S.]. 2011 2011-11-01;85(21):11361–71. 10.1128/JVI.05046-11 21835794 PMC3194980

[49] Goodier JL, Cheung LE, Kazazian HJ. Mapping the LINE1 ORF1 protein interactome reveals associated inhibitors of human retrotransposition. NUCLEIC ACIDS RES. [Journal Article; Research Support, Non-U.S. Gov't]. 2013 2013-8-01;41(15):7401–19. 10.1093/nar/gkt512 23749060 PMC3753637

[50] Wang T, Tian C, Zhang W, Sarkis PT, Yu XF. Interaction with 7SL RNA but not with HIV-1 genomic RNA or P bodies is required for APOBEC3F virion packaging. J MOL BIOL. [Journal Article; Research Support, N.I.H., Extramural; Research Support, Non-U.S. Gov't]. 2008 2008-1-25;375(4):1098–112. 18067920 10.1016/j.jmb.2007.11.017

[51] Bishop KN, Holmes RK, Malim MH. Antiviral potency of APOBEC proteins does not correlate with cytidine deamination. J VIROL. [Journal Article; Research Support, Non-U.S. Gov't]. 2006 2006-9-01;80(17):8450–8. 16912295 10.1128/JVI.00839-06 PMC1563846

